# Harnessing peroxisome proliferator-activated receptor γ agonists to induce Heme Oxygenase-1: a promising approach for pulmonary inflammatory disorders

**DOI:** 10.1186/s12964-024-01501-4

**Published:** 2024-02-15

**Authors:** I-Ta Lee, Chien-Chung Yang, Chuen-Mao Yang

**Affiliations:** 1 School of Dentistry, College of Oral Medicine, Taipei Medical University, Taipei, 110301 Taiwan; 2 Department of Traditional Chinese Medicine, Chang Gung Memorial Hospital at Taoyuan, Taoyuan, 333008 Taiwan; 3 School of Traditional Chinese Medicine, College of Medicine, Chang Gung University, Taoyuan, 333323 Taiwan; 4 Graduate Institute of Biomedical and Pharmaceutical Science, Fu Jen Catholic University, New Taipei City, 242062 Taiwan

## Abstract

The activation of peroxisome proliferator-activated receptor (PPAR)-γ has been extensively shown to attenuate inflammatory responses in conditions such as asthma, acute lung injury, and acute respiratory distress syndrome, as demonstrated in animal studies. However, the precise molecular mechanisms underlying these inhibitory effects remain largely unknown. The upregulation of heme oxygenase-1 (HO-1) has been shown to confer protective effects, including antioxidant, antiapoptotic, and immunomodulatory effects in vitro and in vivo. PPARγ is highly expressed not only in adipose tissues but also in various other tissues, including the pulmonary system. Thiazolidinediones (TZDs) are highly selective agonists for PPARγ and are used as antihyperglycemic medications. These observations suggest that PPARγ agonists could modulate metabolism and inflammation. Several studies have indicated that PPARγ agonists may serve as potential therapeutic candidates in inflammation-related diseases by upregulating HO-1, which in turn modulates inflammatory responses. In the respiratory system, exposure to external insults triggers the expression of inflammatory molecules, such as cytokines, chemokines, adhesion molecules, matrix metalloproteinases, and reactive oxygen species, leading to the development of pulmonary inflammatory diseases. Previous studies have demonstrated that the upregulation of HO-1 protects tissues and cells from external insults, indicating that the induction of HO-1 by PPARγ agonists could exert protective effects by inhibiting inflammatory signaling pathways and attenuating the development of pulmonary inflammatory diseases. However, the mechanisms underlying TZD-induced HO-1 expression are not well understood. This review aimed to elucidate the molecular mechanisms through which PPARγ agonists induce the expression of HO-1 and explore how they protect against inflammatory and oxidative responses.

## Introduction

**Fig. 1:**
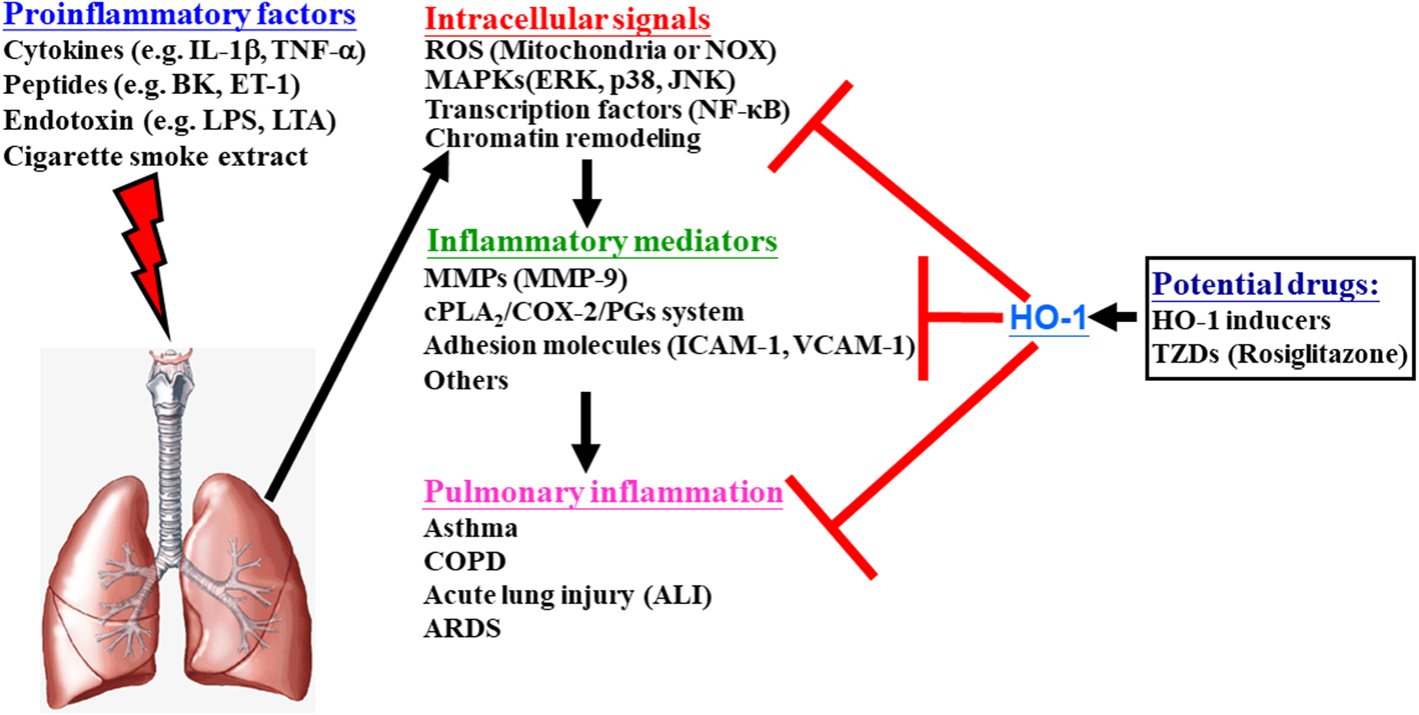
Pathways in pulmonary inflammation and potential therapeutic interventions. During pulmonary inflammation, proinflammatory factors like CSE, ATP, peptides (e.g., bradykinin, BK and *endothelin-1, ET-1*), cytokines (TNF-α and IL-1β), and endotoxins (e.g., LTA and LPS) increase. These factors induce inflammatory mediators (e.g., MMPs, adhesion molecules, COX-2, or cPLA_2_) through various signaling molecules, including mitochondrial or NOX-derived ROS generation, MAPKs activation, transactivation of growth factor receptors, and transcription factors in pulmonary resident cells (alveolar epithelial cells and tracheal smooth muscle cells). These changes lead to pathological alterations in these cells. Furthermore, potential therapeutic drugs such as PPAR agonists might protect against pulmonary inflammation by inducing antioxidant proteins like HO-1. It is hypothesized that these drugs induce HO-1 expression through ROS-dependent signals, directly preventing lung injury and inflammation

Chronic inflammation is at the core of airway and pulmonary inflammatory diseases, including asthma, chronic obstructive pulmonary disease (COPD), acute lung injury, and acute respiratory distress syndrome (ARDS) [1]. Various proinflammatory mediators, such as cigarette smoke extract (CSE), adenosine-5′-triphosphate (ATP), tumor necrosis factor-α (TNF-α), interleukin-1β (IL-1β), lipopolysaccharide (LPS), and lipoteichoic acid (LTA), play pivotal roles in initiating and sustaining inflammation in the pulmonary system [2–10]. These inflammatory responses are primarily triggered in two key types of lung cells. Airway smooth muscle cells, which are typically involved in ventilation regulation, become responsive to various external substances and proinflammatory mediators under pathological conditions such as asthma. Furthermore, alveolar epithelial type II cells, which are vital for gas exchange, are highly susceptible to oxidants. In response to exposure to proinflammatory mediators and oxidative stress, these lung cells release a cascade of cytokines, chemokines, and inflammatory mediators, including IL-1β, TNF-α, cyclooxygenase-2 (COX-2), cytosolic phospholipase A_2_ (cPLA_2_), and matrix metalloproteinase-9 (MMP-9) [11]. Previous studies have revealed the induction of proteins such as COX-2/prostaglandin E_2_ (PGE_2_) [2, 6, 12, 13], cPLA_2_ [8, 14, 15], MMP-9 [3, 16, 17], intercellular adhesion molecule-1 (ICAM-1) [7, 18, 19], and vascular cell adhesion molecule-1 (VCAM-1) [5, 19–21] and reactive oxygen species (ROS) [7, 21, 22] in response to various stimuli and pathogens associated with pulmonary inflammation (Fig. 1). The induction of these factors is likely mediated by NADPH oxidase (NOX)/ROS, intracellular signaling pathways, and transcription factors, illustrating the complex interplay between ROS and the expression of inflammatory proteins induced by proinflammatory mediators (Fig. 1). Consequently, understanding these mechanisms is crucial for developing therapeutic strategies for pulmonary inflammatory diseases. Oxidative stress plays a pivotal role in pulmonary inflammation. Early interventions targeting oxidative stress could delay the onset and progression of inflammation. Despite extensive research, an effective therapeutic strategy to prevent pulmonary inflammation progression remains elusive. Therefore, the development of antioxidative and anti-inflammatory drugs has emerged as a promising avenue to inhibit the pathological progression of these diseases.

Peroxisome proliferator-activated receptor (PPAR) agonists, which are widely used in clinical settings, exert multiple effects, including anti-inflammatory and antioxidative effects, on various tissues and organs. PPARγ is abundantly expressed in human adipose tissue, and its expression levels are comparatively lower in bone marrow, skeletal muscle, liver, heart, and stromal cells [23, 24]. PPARγ plays a crucial role in glucose metabolism, adipocyte differentiation, and the inflammatory response and is important in various physiological processes. Its therapeutic applications extend to the treatment of conditions such as diabetes, fatty liver disease, cardiovascular disease, and neuroinflammation [25–30]. PPAR agonists, while exhibiting increased therapeutic efficacy and improved glycemic control, are part of a complex clinical landscape due to a spectrum of associated adverse reactions. Notably, these adverse events include an increased risk of cardiovascular events, including congestive heart failure, cardiovascular death, and myocardial infarction [31–33]. Additionally, users may face increased risk of fractures, bladder cancer, and macular edema and weight gain [31–33]. It is imperative to approach the clinical use of PPAR agonists with a nuanced understanding of the potential hepatotoxicity associated with their use [31–33]. This consideration of therapeutic benefits and associated risks underscores the importance of careful patient selection and monitoring when using PPAR agonists in various medical contexts. By leveraging these pleiotropic effects, the use of PPAR agonists is a potential strategy for managing pulmonary inflammatory diseases (Fig. 1). Further research and clinical exploration in this direction may lead to innovative treatments for these challenging conditions.

## The anti-inflammatory and antioxidative effects of PPAR agonists in diverse inflammatory disorders

Recently, research has identified three distinct PPAR isoforms (PPARα, PPARβ, and PPARγ), which are encoded by unique genes. Notably, two PPARγ isoforms are generated through alternative splicing of exons A1, A2, and B1, giving rise to γ1, γ2, and γ3 variants, respectively [34]. These PPARs are widely expressed in various animal and human tissues. In adult rodents, PPARα is prominently found in tissues with high peroxisome-dependent activities or rapid fatty acid catabolism, such as hepatocytes, cardiomyocytes, proximal tubules of the kidney cortex, the intestinal mucosa, and brown adipose tissue [35, 36]. In humans, PPARα is notably expressed in skeletal muscle, heart, liver, kidney, intestine, and pancreas. Additionally, it is present, albeit at lower levels, in adipose tissue, lung, and placenta [23]. PPARα’s primary functions include the regulation of fatty acid oxidation and lipoprotein metabolism and involvement in anti-inflammatory/antioxidant pathways. PPARβ, on the other hand, is ubiquitously expressed in most rodent tissues and in higher quantities than PPARα and PPARγ [37]. This widespread presence extends to human tissues, making PPARβ a key player in fatty acid metabolism regulation and the suppression of macrophage-mediated inflammation. PPARγ, which is prominently expressed in adipose tissue and moderately in the large intestine and select immune system components such as the spleen in rodents, is abundantly found in human adipose tissue. However, in humans, it is expressed at lower levels in bone marrow, skeletal muscle, liver, heart, and stromal cells [23, 24]. PPARγ plays a pivotal role in glucose metabolism, adipocyte differentiation, and inflammatory responses.

Natural ligands of PPARs include fatty acids and eicosanoids. Leukotriene B4 activates PPARα, while prostaglandin J_2_ (PGJ_2_) serves as a ligand for PPARγ. Additionally, synthetic ligands such as antidiabetic glitazones and hypolipidemic fibrates activate PPARγ and PPARα, respectively. Research has shown that PPARα regulates the oxidation of fatty acids in the liver by controlling genes containing peroxisome proliferator response elements (PPREs) in their promoter regions [38]. Mice lacking PPARα show prolonged responses to inflammatory mediators. Furthermore, PPAR agonists inhibit the activation of genes associated with the inflammatory response, such as IL-2, IL-6, IL-8, TNF-α, and metalloproteases, by modulating signaling pathways involving nuclear factor κ-light-chain-enhancer of activated B cells (NF-κB), activator protein 1 (AP-1), and signal transducer and activator of transcription proteins (STATs) [39]. The activation of PPARγ also mediates adipogenesis; studies have demonstrated that synthetic PPARγ ligands such as rosiglitazone [40] and natural ligands such as 15d-PGJ_2_ [41] reduce serum levels and transcription of TNF-α.

In summary, PPARs play multiple roles in the β-oxidation of fatty acids and the metabolism of arachidonic acid metabolites. Beyond these functions, they exhibit significant anti-inflammatory potential. These findings strongly suggest that PPARs are key regulators that control inflammation, paving the way for potential therapeutic interventions in inflammatory diseases.

## Elucidating the mechanisms by which PPARγ agonists induce heme oxygenase-1 (HO-1) expression

PPARs hold significant promise as therapeutic agents for treating lung inflammation [42, 43]. In particular, PPARγ ligands can inhibit the release of proinflammatory cytokines from airway epithelial cells and reduce airway hyperresponsiveness in murine models of asthma [44]. A noteworthy example is rosiglitazone, a PPARγ agonist that is known not only for its role in maintaining glucose and lipid homeostasis but also for its capacity to attenuate airway inflammation through the upregulation of HO-1 [45]. HO-1, which is a direct transcriptional target of PPARγ, exhibits potent anti-inflammatory, antioxidant, and apoptosis-regulating properties [46, 47]. Furthermore, the upregulation of HO-1 by PPARγ agonists has been linked to the inhibition of pulmonary cell proliferation and remodeling [48]. However, the precise mechanisms governing rosiglitazone-induced PPARγ/HO-1 expression in human pulmonary alveolar epithelial cells (HPAEpiCs) remain unclear. In the following sections, we will delve into the molecular mechanisms through which PPARγ agonists induce HO-1 expression. We will examine the activation of various signaling components and transcription factors that contribute to this regulatory process. This understanding will not only shed light on the potential therapeutic applications of PPARγ agonists in managing pulmonary inflammation but also unveil the underlying molecular mechanisms that govern these therapeutic effects.

### NOX and HO-1 expression

ROS are pivotal messengers during normal physiological functions, and their effects oscillate between beneficial and inflammatory, depending on their concentrations [49]. HO-1, which is a sentinel enzyme, is activated in response to intracellular oxidative stress or disruptions in intracellular reduction–oxidation (redox) equilibrium, serving as a protector against inflammatory responses [50]. Notably, activated NOX is a prominent source of ROS, which act as secondary messengers that induce the expression of HO-1, a potent defense mechanism against inflammation [51–53]. Many stimuli can induce NOX/ROS-dependent HO-1 expression, underscoring the complexity of this regulatory pathway [54, 55]. Intriguingly, PPAR agonists activate protein kinases through ROS-dependent pathways, thereby regulating a multitude of genes, including HO-1 [56, 57]. Furthermore, endogenous PPARγ ligands such as 15d-PGJ_2_ have been shown to induce ROS generation, stimulating the expression of HO-1 via the nuclear factor E2-related factor 2 (Nrf2) pathway [58]. PPARγ ligands, with their multifaceted effects, have been shown to inhibit airway inflammation and hyperresponsiveness through PPARγ-independent mechanisms in mouse models challenged with allergens [59]. The NOX/ROS system, which is a complex web of signaling components, induces HO-1 expression through a choreographed activation process. In response to stimulation, cytosolic regulatory subunits such as p40^phox^, p47^phox^, p67^phox^, Ras-related C3 botulinum toxin substrate (Rac)1, and Rac2 translocate to the membrane. There, they assemble with membrane-bound components, including gp91^phox^/p22^phox^, leading to the generation of ROS (O_2_^•−^/H_2_O_2_) [60]. Specifically, NOX2, which is a prominent NOX enzyme in pulmonary alveolar epithelial cells, recruits p22^phox^ and p47^phox^, thereby producing O_2_^•−^ [60]. Phosphorylation of p47^phox^ is a pivotal event that is essential for NOX activation and ROS generation [61]. In our studies of HPAEpiCs, we discovered a significant connection. Rosiglitazone, which is a PPARγ agonist, upregulates HO-1 through NOX-dependent ROS generation [62]. This relationship was underscored by the attenuation of rosiglitazone-induced HO-1 expression when p47^phox^ or NOX was inhibited, as well as in the presence of ROS scavengers. Moreover, NOX inhibitors exerted their effects by preventing p47^phox^ phosphorylation or membrane translocation, effectively inhibiting NOX activation and ROS generation [63]. These findings emphasize the vital role of NOX/ROS signaling in the responses induced by rosiglitazone, further illuminating the underlying mechanisms by which PPARγ agonists protect against pulmonary inflammation. However, the precise mechanisms by which rosiglitazone triggers the phosphorylation of p47^phox^ and the recruitment of NOX subunits to the plasma membrane, ultimately leading to ROS generation, remain unknown. Further investigations will unveil critical insights, paving the way for a deeper understanding of these complex molecular interactions.

### Protein tyrosine kinases and HO-1 expression

Protein tyrosine kinases are pivotal players in intracellular signaling and are categorized as receptor tyrosine kinases (e.g., EGFR and PDGFR) and nonreceptor tyrosine kinases (e.g., c-Src and Pyk2). These kinases modulate diverse cellular functions. Notably, NOX/ROS production activates downstream components such as c-Src and Pyk2, influencing cellular activities [64, 65]. Specifically, c-Src acts as an upstream regulator, phosphorylating Pyk2, which is stimulated by thiazolidinediones (TZDs) and regulated by ROS. This phosphorylation event triggers HO-1 expression, protecting various cell types from oxidative damage [65–69]. Our investigations of HPAEpiCs showed that rosiglitazone-induced HO-1 expression involved the phosphorylation of c-Src and Pyk2. The nonreceptor tyrosine kinases c-Src, and Pyk2 are pivotal players in the protective effects of rosiglitazone against pulmonary inflammation [70]. ROS-dependent phosphorylation of c-Src and Pyk2 is critical for HO-1 induction by rosiglitazone, offering a promising avenue for therapeutic interventions against pulmonary inflammation. Notably, c-Src and Pyk2 are distinct from receptor tyrosine kinases such as EGFR and PDGFR. Hyperoxia-induced phosphorylation of EGFR has been shown to induce HO-1 expression in pulmonary epithelial cells [71]. This intriguing interplay between PPARγ agonists and receptor tyrosine kinases such as EGFR and PDGFR presents a compelling area for future exploration. Understanding whether rosiglitazone stimulates the phosphorylation of EGFR or PDGFR, thereby triggering HO-1 expression in pulmonary resident cells, holds the key to unraveling another layer of the complex molecular landscape underpinning the protective effects of PPARγ agonists against pulmonary inflammation. In essence, the interplay between nonreceptor tyrosine kinases, receptor tyrosine kinases, and PPARγ agonists in the regulation of HO-1 expression opens up exciting avenues for further research. These findings not only deepen our understanding of the molecular mechanisms but also offer potential targets for therapeutic strategies to mitigate pulmonary inflammation.

### Protein kinase Cs (PKCs) and HO-1 expression

The PKC family is a diverse group of at least eleven isoforms that are categorized into three groups and are finely tuned to cellular signals for precise regulation [72]. Notably, classical PKCs such as PKCα, β/δ, and γ are activated by diacylglycerol (DAG) in a calcium-dependent manner, serving as pivotal players in various pathophysiological responses in different cellular contexts [73]. These serine- and threonine-specific protein kinases are fundamental to multiple physiological processes and gene expression in various cellular contexts [74]. Their activation results in the phosphorylation of specific target proteins, which is involved in various pathological conditions, including pulmonary inflammatory diseases [75]. Furthermore, PKC isoforms modulate both pro- and anti-inflammatory systems, underscoring their vital roles in cellular functions and gene regulation [76]. Numerous studies have demonstrated the regulatory role of PKCs, especially PKCα, in orchestrating the expression of HO-1 in response to diverse stimuli in many cell types [77, 78]. Specifically, PKCα actively participates in immune signaling pathways, influencing the expression of inflammatory genes [79, 80]. By enhancing the phosphorylation of several protein kinases, PKCα facilitates the expression of specific proteins, which has been extensively observed in various tissues and cells [79]. Furthermore, the PKCα/Erk1/2 pathway has been identified as a key regulator of HO-1 expression induced by compounds such as curcumin [81]. Our research substantiates these findings. Using small interfering RNA (siRNA) to silence PKCα significantly decreases rosiglitazone-induced HO-1 expression in HPAEpiCs through PKCα phosphorylation [70]. Moreover, PKCα plays a crucial role in the Nrf2/HO-1 pathway by protecting against oxidative stress [82]. Collectively, our results underscore the pivotal role of phosphorylated PKCs, particularly PKCα, in mediating rosiglitazone-induced HO-1 expression, offering promising avenues for therapeutic interventions against pulmonary inflammatory diseases. However, unraveling the effects of other PKC isoforms on HO-1 expression induced by TZDs is a critical area that requires further investigation.

### AMPKα and HO-1 expression

5′-Adenosine monophosphate-activated protein kinase α (AMPKα) is a crucial cellular sensor that is activated in response to reduced ATP levels and plays a pivotal role in cellular homeostasis. This enzyme not only enhances cyclic adenosine monophosphate (cAMP) production, protecting endothelial cells against proliferation and angiogenesis [83], but also orchestrates a myriad of cellular processes through the phosphorylation of target proteins and the regulation of gene expression. Notably, AMPKα is a potent inducer of Nrf2/HO-1 gene expression, effectively inhibiting inflammatory responses in animal models [84]. The multifaceted role of AMPKα in immune regulation is underscored by its ability to induce the production of IL-10, negatively regulate IκBα degradation, inhibit glycogen synthase kinase (GSK) 3β, and activate Akt and cAMP response element-binding protein (CREB). These actions culminate in the promotion of an anti-inflammatory phenotype, indicating that AMPKα is a key player in immune modulation [85]. Moreover, AMPKα’s influence extends to airway inflammation, where it uses multiple pathways to suppress the release of proinflammatory cytokines and inhibit the NF-κB pathway, thereby mitigating pulmonary inflammation and emphysema in lung disease models [86, 87]. The protective and anti-inflammatory effects of AMPKα are often mediated by the induction of HO-1 expression in lung epithelial cells, which serves as a linchpin in the cellular defense against inflammatory insult [88, 89]. In the specific context of our study, we discovered that rosiglitazone activated AMPKα, leading to the induction of HO-1 expression and subsequent anti-inflammatory effects on HPAEpiCs [70]. This finding highlights the pivotal role of AMPKα phosphorylation in mediating the protective effects of rosiglitazone against pulmonary inflammatory diseases. Furthermore, the intriguing prospect that AMPKα influences downstream components, particularly mitogen-activated protein kinases (MAPKs), in the context of HO-1 expression opens a promising avenue for future research. Understanding the interplay between AMPKα and MAPKs could lead to the discovery of novel therapeutic strategies, potentially revolutionizing our approach to combating pulmonary inflammatory disorders [90, 91]. This network of signaling pathways presents an exciting frontier for exploration, promising not only deeper insights into cellular responses but also innovative therapeutic interventions for inflammatory diseases.

### MAPKs and HO-1 expression

MAPKs form a pivotal signaling network that regulates many cellular functions, including proliferation, apoptosis, motility, differentiation, immunity, and responses to oxidative stress. MAPKs include three distinct groups in mammalian cells: extracellular signal-regulated protein kinases (ERKs: ERK1/2), c-Jun NH2-terminal kinases (JNKs: JNK1, JNK2, and JNK3), and p38 MAP kinases (p38α, p38β, p38γ, and p38δ). In the context of HO-1 regulation, MAPK pathways are conduits through which various stimuli induce HO-1 expression via downstream protein kinases and transcription factors [92–95]. Interestingly, HO-1 induction is linked to the activation of MAPKs mediated by protein phosphorylation and redox reactions. Sodium arsenite triggers HO-1 expression by activating JNK1/2 in rat hepatocytes, while in chicken hepatoma cells, arsenite uses both the ERK1/2 and p38 MAPK pathways to induce HO-1 expression [96, 97]. The ischemia–reperfusion lung model implicates JNK1/2 and p38 MAPK in HO-1 expression [98], and in various cell types, p38 MAPK activation upregulates HO-1, as observed in porcine renal epithelial cells and human hepatoma cells stimulated by curcumin [93, 99]. Furthermore, 15d-PGJ_2_, which is an endogenous PPARγ agonist, induces HO-1 expression through p38 MAPKα in rat vascular smooth muscle cells [100]. Consistent with these findings, our research uncovered the involvement of p38 MAPKα phosphorylation in rosiglitazone-induced HO-1 expression in HPAEpiCs [70]. These observations underscore the significant role of MAPKs, including p38 MAPKα, in TZD-induced HO-1 expression. However, the distinct roles of ERK1/2 and JNK1/2 in rosiglitazone-induced HO-1 expression in HPAEpiCs need further exploration. This interplay between different MAPK pathways and HO-1 induction is critical for future investigations, offering potential insights into cell-specific responses and paving the way for novel therapeutic interventions in inflammatory diseases.

### Phosphoinositide 3-kinase/Akt (PI3K/Akt) and HO-1 expression

The PI3K/Akt pathway, which is a pivotal antiapoptotic survival pathway, is regulated by various receptor-dependent mechanisms that are activated by growth factors and cytokines [101]. Notably, in models featuring PI3K genetic deficiency, this kinase has been implicated in the regulation of inflammatory reactions, emphasizing its importance in cellular responses [102]. A growing body of evidence underscores the multifaceted role of PI3K/Akt in HO-1 regulation. Activation of PI3K/Akt not only upregulates HO-1 gene expression but also connects the protective effects of this signaling cascade to the beneficial effects of HO-1 [103]. Immune cells exhibit upregulated HO-1 gene expression mediated by PI3K/Akt activation in response to diverse stimuli [103–105]. Intriguingly, HO-1 induction has been linked to the activation of PI3K/Akt through a mitochondrial redox-dependent pathway in vascular endothelial cells, highlighting the nuanced regulatory mechanisms involved in HO-1 expression [106]. Furthermore, NOX/ROS-dependent HO-1 expression has been shown to be mediated by PI3K/Akt activation, resulting in protection against oxidative stresses [107, 108]. Activated Akt, in turn, triggers the expression of Nrf2 and subsequently HO-1, increasing cellular defense mechanisms and promoting cell survival in response to stresses [107, 108]. Consistent with these findings, our research showed the critical role of Akt phosphorylation induced by rosiglitazone in facilitating HO-1 expression, and this effect was significantly attenuated by LY294002 treatment or transfection with Akt siRNA in HPAEpiCs [62]. This discovery underscores the pivotal role of PI3K/Akt in TZD-induced HO-1 expression in many cell types. However, the regulatory mechanisms of PI3K/Akt-mediated HO-1 induction in response to PPARγ agonists remain unclear. Delving deeper into these mechanisms will not only enhance our understanding of the complexities of cellular signaling pathways but may also lead to novel therapeutic strategies to protect against inflammatory diseases.

### Janus kinase (JAK)/signal transducer and activator of transcription 3 (STAT3) and HO-1 expression

The intricacies of the JAK/STAT3 signaling cascade, which links HO-1 gene expression to cytokines and oxidative stress, remain unclear. However, the involvement of STAT3 activation in this regulatory pathway has been suggested based on existing evidence [109]. The JAK/STAT signaling pathway plays a pivotal role in the immune system, serving as a major mediator of cytokine-activated pathways. In endothelial cells, STAT3 has been shown to mediate HO-1-dependent protection against hyperoxic lung injury, underscoring its importance in cellular defense mechanisms [110]. Functional STAT3 elements have been identified in the promoter regions of both rat [111] and human [112] HO-1 genes, indicating a direct regulatory role of STAT3 in HO-1 expression. In rat hepatocytes, IL-6-induced HO-1 gene expression has been shown to be mediated by the JAK/STAT3 pathway, highlighting the interplay between this signaling cascade and HO-1 induction [111]. Notably, the specific interactions between JAK/STAT signaling and HO-1 expression in HPAEpiCs remain unexplored, representing a significant gap in our understanding. Therefore, the potential regulatory role of JAK/STAT in HO-1 gene expression induced by PPARγ agonists, particularly in pulmonary resident cells such as HPAEpiCs, holds substantial promise for further investigation. Exploring this aspect will not only broaden our understanding of the molecular mechanisms of HO-1 regulation but also open avenues for targeted therapeutic interventions against inflammatory diseases.

### Transcriptional regulation of HO-1 gene expression: key transcription factors and their functions

Understanding the machinery of HO-1 expression is crucial for targeted anti-inflammatory treatments. HO-1, which is a versatile enzyme, is triggered by various stimuli, such as oxidative stress, cytokines, bacterial compounds, and growth factors. Its expression is primarily governed at the transcriptional level, and multiple cis-acting regulatory elements within the HO-1 promoter control its basal and inducible expression in different species [104, 113, 114]. Within the promoter regions of HO-1, two upstream enhancer regions, namely, E1 and E2, play pivotal roles in redox-dependent induction [113]. These enhancer regions house several antioxidant response elements (AREs) [115], which are also found in other stress-inducible antioxidant and phase 2 detoxifying gene promoters [116, 117]. Notably, a GT-microsatellite polymorphism in the proximal human HO-1 gene promoter distinguishes it from its rodent counterpart. This polymorphism, which is characterized by varying numbers of GT repeats, significantly impacts HO-1 inducibility; fewer GT repeats correlate with increased HO-1 expression in response to stressors. Individuals harboring this allele are protected against cardiovascular disorders [118, 119]. In this comprehensive review, we focus on the pivotal roles of major transcription factors such as Nrf2, silent information regulator type-1 (SIRT1)/peroxisome proliferator-activated receptor gamma coactivator 1-α (PGC1α), PPARγ, Sp1, and AP-1 in the regulation of HO-1 gene expression, specifically in response to PPARγ agonists. Delving into these regulatory pathways will not only enhance our understanding of HO-1 modulation but also pave the way for refining anti-inflammatory therapeutic strategies.

#### Decoding HO-1 expression: unraveling coordinated activation via the Nrf2 signaling pathway

The regulation of HO-1, a critical player in oxidative stress management, is governed by a complex network involving multiple factors and pathways. At the heart of this system is Nrf2, which is essential for the transcriptional regulation of the *hmox1* gene that encodes HO-1 [50, 51]. Normally, Nrf2 is restrained in the cytoplasm by its interaction with Kelch-like ECH-associated protein (Keap1). However, when cells encounter oxidative stress, marked by an increase in ROS, this interaction is disrupted [51]. As a result, Nrf2 is released and accumulates in the nucleus, where it forms dimers with small Maf proteins and binds to AREs in the promoters of genes like *hmox1*, driving the transcription of genes involved in antioxidant defense [120–122]. This response to oxidative stress serves to bolster the cell’s antioxidant capacity, with HO-1 being a key component. Alongside Nrf2, the heme-binding protein BTB and CNC homologue 1 (Bach1), which negatively regulates *hmox1* transcription, is also involved [123]. Bach1 competes with Nrf2 for binding sites on DNA. Interestingly, heme influences this interaction; it not only promotes the release of Nrf2 from Keap1 but also aids in the export of Bach1 from the nucleus, thereby facilitating Nrf2’s activity [124].

The role of the PPARγ pathway in regulating HO-1 is another crucial aspect. PPARγ agonists, such as rosiglitazone, have been shown to upregulate HO-1 expression. This not only aids in maintaining redox balance but also exhibits anti-inflammatory effects, suggesting a therapeutic potential in conditions characterized by oxidative stress. Our research has highlighted that rosiglitazone can induce HO-1 expression in HPAEpiCs through mechanisms that are both dependent on and independent of PPARγ. This includes the activation of PPARγ leading to increased HO-1 expression, which in turn helps reduce inflammation and tissue remodeling in the airways [45]. Interestingly, this process seems to be unaffected by factors such as NOX/ROS, c-Src/Pyk2, Akt, and even Nrf2 itself [70]. Further, we found that rosiglitazone’s stimulation of HO-1 expression involves the activation of Nrf2 via a pathway that does not rely on PPARγ. This was evident from the increased binding of Nrf2 to the ARE in the HO-1 promoter, a process that was sensitive to Nrf2 siRNA and pharmacological inhibitors but not to PPARγ antagonists. Additionally, a decrease in Keap1 levels corresponded with an increase in HO-1 protein in HPAEpiCs, highlighting the significant role of Nrf2 in mediating the response to rosiglitazone.

This intricate interplay between various factors emphasizes the delicate balance between prooxidant forces and antioxidant defenses in the cell. The upregulation of HO-1 is a key protective mechanism against oxidative stress, but the effectiveness of this response is dependent on the specific conditions of stress, including its intensity and duration. This underscores the need for further research to explore the dual PPARγ-dependent and independent mechanisms involved in rosiglitazone-induced HO-1 expression and to understand their wider implications in the management of oxidative stress.

#### Regulation of HO-1 expression by SIRT1/PGC1α

SIRT1, which is a member of the NAD-dependent deacetylases known as sirtuins, plays a pivotal role in anti-inflammatory responses by suppressing the production of various proinflammatory cytokines [125, 126]. Recent research highlights the protective effects of SIRT1-mediated HO-1 induction, which not only suppresses inflammatory responses but also activates antiapoptotic pathways [127]. The activation of SIRT1, which is often facilitated by compounds such as resveratrol, effectively inhibits bronchial inflammation induced by cigarette smoke in the lungs [128, 129]. Additionally, SIRT1 prevents multiple inflammatory responses by enhancing PGC1α activation and downregulating NF-κB [130]. Overexpression of SIRT1 reduces the acetylation levels of PGC1α, thereby protecting against neuroinflammation [131]. Nuclear receptors interact with coactivators such as PGC1α and corepressors such as NCoR. PGC1α overexpression has been shown to protect endothelial cells against oxidative stress [132]. In contrast, NCoR not only prevents inflammatory target gene expression in the absence of stimuli but is also essential for PPAR ligand-dependent transcription [133]. SIRT1’s interaction with NCoR could negatively regulate PPARγ when PGC1α levels are low [134]. Our observations align with these findings. Rosiglitazone induces the phosphorylation of SIRT1 specifically in the cytosol, facilitating the deacetylation of Ac-PGC1α. This process promotes the translocation of PGC1α from the cytosol to the nucleus, where it interacts with PPARγ [70]. Furthermore, there is a reduction in NCoR levels in the nucleus, enhancing the phosphorylation of PPARγ. This interplay between the transcriptional corepressor NCoR and the coactivator PGC1α underscores their involvement in rosiglitazone-induced HO-1 expression through a PPARγ-dependent pathway in HPAEpiCs [70].

#### Regulation of HO-1 expression by PPARγ: insights and mechanisms

PPARγ belongs to the nuclear hormone receptor family and functions as a transcriptional activator, relying on ligand binding to activate its domain. All PPAR members share similar structural domains: a ligand-dependent activation domain, a DNA-binding domain, and an amino-terminal region enabling ligand-independent activation [34]. PPAR-mediated transactivation occurs when PPAR and 9-cis retinoic acid receptor (RXR) bind to PPAR-response elements (PPREs) in response to ligand activation. Ligand binding induces conformational changes, facilitating coactivator recruitment and releasing corepressors such as NCoR. Ligand binding or other activation processes, including phosphorylation, induce conformational shifts in PPAR, particularly in the ligand-binding domain (LBD) and the C-terminal helix activation function-2 (AF-2). This alteration generates new protein–protein interaction surfaces, allowing the recruitment of specific coactivators such as steroid receptor coactivator 1 (SRC-1), CREB-binding protein (CBP)/p300, and PGC-1. Subsequently, the regulatory effects of this complex are transduced to the basal transcriptional machinery [34]. Phosphorylation at the Ser^112^ residue of PPARγ has been shown to influence its binding affinity with PPARγ ligands and its interactions with coactivators and posttranslational events [135, 136]. Notably, rosiglitazone protects against acute lung injury and inflammation through HO-1 expression [137, 138]. Consistent with these findings, our study reveals that rosiglitazone-induced HO-1 expression occurs through a PPARγ-dependent mechanism in HPAEpiCs [62]. However, the interactions among various nuclear components that initiate HO-1 gene expression are highly complex and require further clarification.

#### Regulation of HO-1 expression by the transcription factor Sp1

Sp1, which is a versatile transcription factor, responds to many signals, including oxidative stress [139, 140]. This factor affects vital physiological processes, controlling cell cycle dynamics, growth modulation, hormonal responses, apoptosis, and angiogenesis [141]. Beyond self-regulation, Sp1 governs genes that are essential for cellular homeostasis, forging direct connections with critical elements such as TATA-binding protein-associated factors [142], cAMP response domains [143], NF-κB [144], and vascular endothelial growth factor receptor-2 [145]. This regulatory role is modulated by dynamic modifications, such as phosphorylation, acetylation, and methylation, which fine-tune Sp1 protein levels, transactivation potential, and DNA binding affinity [139, 146]. In the context of PPARγ agonist-induced responses, Sp1 is a linchpin, orchestrating the complex events that culminate in HO-1 induction in pulmonary resident cells. The involvement of Sp1 indicates a sophisticated regulatory network in which Sp1 acts as a pivotal mediator, coordinating PPARγ signaling. Unraveling Sp1’s role in this context would not only shed light on fundamental molecular mechanisms but also holds promise for the development of therapeutic strategies for treating diverse pulmonary disorders.

#### The involvement of AP-1 in HO-1 expression

The AP-1 transcription factor complex, which is composed of members of the Jun (c-Jun, JunB, JunD), Fos (c-Fos, FosB, Fra1, Fra2), and activating TF (ATF) families, has emerged as a central player in cellular signaling, orchestrating various responses to oxidative and inflammatory stimuli [147, 148]. Activation of AP-1, which is induced by prooxidant and proinflammatory triggers, influences cellular outcomes. Notably, AP-1’s involvement in the induction of the mouse HO-1 gene underscores its role in stress-responsive gene expression, indicating its importance in cellular stress adaptation [113, 149]. This process involves cooperative interactions between AP-1 and other essential transcription factors, such as USF2 and Sp1, which are bound to the regulatory regions of the HO-1 promoter [150, 151]. This interplay emphasizes the multifaceted nature of inducer-dependent HO-1 expression. In the context of pulmonary biology, where oxidative stress and inflammation converge, deciphering the nuanced connections between AP-1 and PPARγ agonists is of paramount importance. These interactions, which affect HO-1 regulation, offer promising avenues for novel therapeutic interventions. Exploring the intricacies of AP-1-mediated HO-1 expression would not only advance our understanding of cellular stress responses but also identify potential strategies for mitigating oxidative stress-related disorders within the pulmonary milieu.

## The positive impact of PPARγ agonists on inflammatory lung disorders

**Fig. 2:**
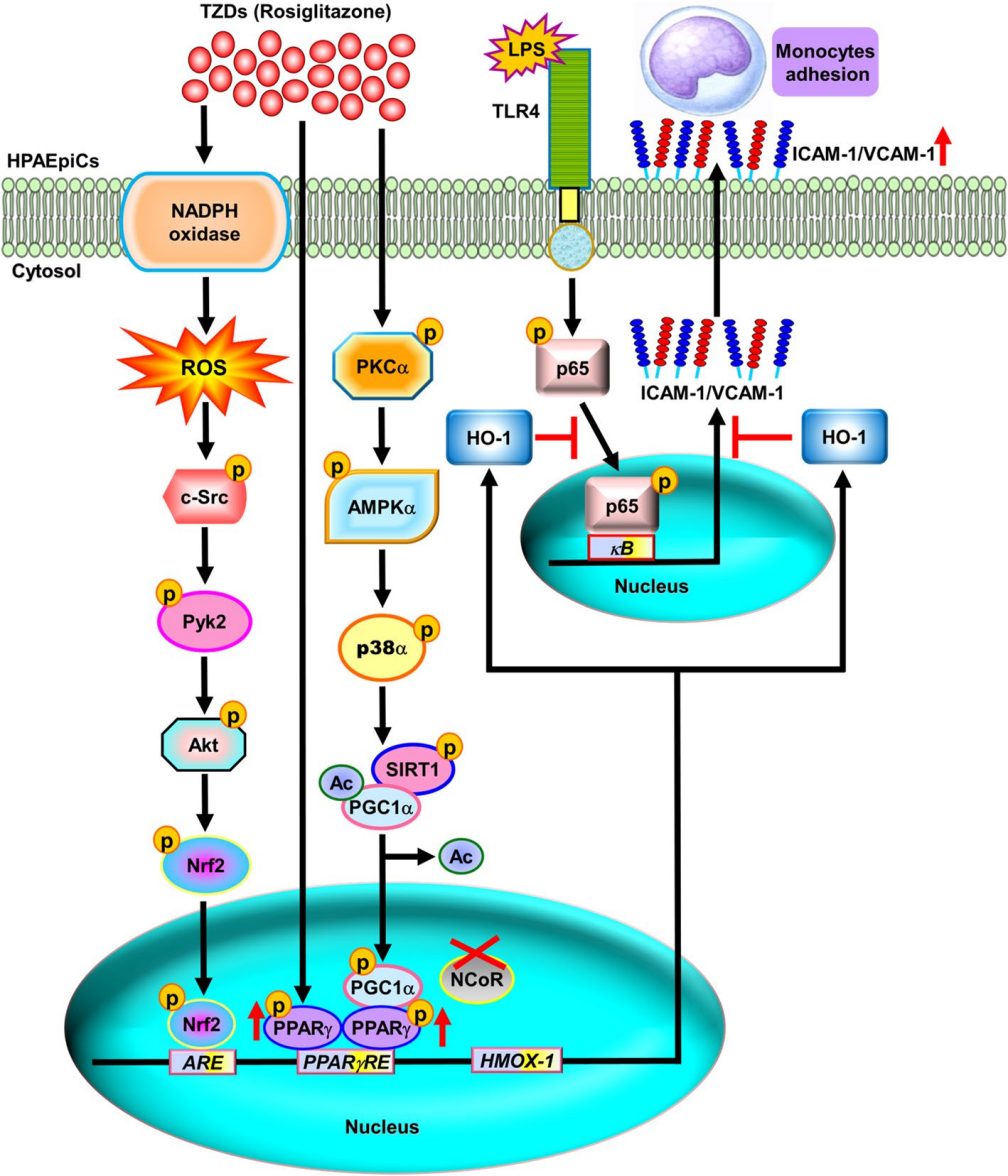
Schematic pathways for rosiglitazone-induced HO-1 expression in HPAEpiCs. Rosiglitazone triggers HO-1 expression in HPAEpiCs through two distinct pathways: PPARγ-dependent and PPARγ-independent mechanisms. PPARγ-dependent pathway: rosiglitazone enhances HO-1 expression by activating a series of events, including PKCα, AMPKα, p38 MAPKα, SIRT1, Ac-PGC1α deacetylation, NCoR fragmentation, and direct binding of activated PPARγ to the HO-1 promoter’s responsive element. PPARγ-independent pathway: in this pathway, rosiglitazone-induced HO-1 expression occurs via NOX/ROS-dependent phosphorylation of c-Src/Pyk2/Akt, leading to Nrf2 activation. Nrf2 then binds to the ARE region of the HO-1 promoter, stimulating HO-1 expression. Upregulation of HO-1 exerts anti-inflammatory effects, particularly on the expression of adhesion molecules like ICAM-1 and VCAM-1, associated with monocyte/leukocyte accumulation in pulmonary resident cells challenged with LPS. These pathways are adapted from prior studies [62, 70] and represent crucial mechanisms in mitigating inflammation in the lungs

In the past decade, evidence has emerged regarding the potential advantages of PPARγ agonists in treating various pulmonary inflammatory diseases. A cohort study conducted by Rinne et al. examined the impact of TZDs on COPD exacerbation in US veterans with COPD and diabetes [132]. Their research revealed a decrease in the risk of COPD exacerbation in patients treated with TZDs, and the incidence rate ratio was 0.86 (95% CI: 0.81–0.92) compared to those using non-TZDs [152]. Another study by the same team focused on diabetic veterans with asthma comorbidity and showed a significant reduction in the risk of asthma exacerbation in patients exposed to TZDs (OR = 0.79, 95% CI: 0.62–0.99) compared to those not exposed to TZDs. Moreover, patients in good compliance with diabetes medications experienced an even more substantial reduction in the risk of asthma exacerbation (OR = 0.64, 95% CI: 0.47–0.85) [153]. These findings underscore the substantial reduction in the risk of COPD and asthma exacerbation in diabetic patients with COPD and asthma due to TZD administration. The consistent observation of the beneficial effects of TZDs in reducing severe COPD exacerbation is evident in the two most recent observational studies [154, 155]. Furthermore, there was a notable decrease in PPAR-gamma gene expression and a median decrease of 45% (*P* = 0.008) observed from baseline to postchallenge in the bronchoalveolar lavage (BAL) fluid of allergic asthma patients. It is important to highlight that this decrease was not evident in the healthy control group [156]. Another study involving smokers with asthma demonstrated that rosiglitazone improved lung function, particularly in forced expiratory volume in 1 s (FEV(1)), compared to the effects of inhaled beclometasone dipropionate [157]. In contrast to these positive results, it is imperative to shed light on findings that deviate from the expected outcomes. For instance, Richards et al. conducted a single-center, double-blind, randomized, placebo-controlled, two-period crossover study exploring the effects of rosiglitazone, which is a PPARγ agonist, on the late asthmatic reaction in an allergen challenge model of asthma [158]. Surprisingly, their results showed only a modest (15%) reduction, which did not reach statistical significance. We acknowledge that several factors, including the duration of the washout period, the dose of rosiglitazone (4 mg), and the severity of the patient conditions, may have contributed to this nonsignificant outcome. This divergence from the expected positive outcome prompts a more critical examination of the nuances surrounding the experimental design and patient characteristics. However, despite these positive outcomes, the mechanisms by which PPARγ agonists affect lung inflammation remain unclear. Numerous studies have shown the anti-inflammatory effects of PPARγ agonists on lung tissues. For example, in a mouse model of asthma induced by ovalbumin (OVA) inhalation, PPARγ agonists such as rosiglitazone and pioglitazone reduced the increases in IL-17 mRNA and protein expression, airway inflammation, and bronchial hyperresponsiveness. Additionally, these agents abrogated increase in NF-κB activity in this asthma model [159]. Another study demonstrated that PPARγ ligands such as rosiglitazone and pioglitazone reduced TNF-α and CC chemokine ligand-5 in the alveolar macrophages of COPD patients. Rosiglitazone also increased the gene expression of M2 macrophages and facilitated the clearance of apoptotic airway neutrophils in an in vivo model of pulmonary inflammation [160]. Moreover, rosiglitazone and SB219994 could inhibit airway neutrophilia induced by LPS and associated chemoattractants (keratinocyte-derived chemokine and granulocyte-colony-stimulating factor) in an animal model [161]. A previous study suggested that a reduction in the expression of PPAR-γ protein in the lungs could lead to pulmonary inflammation and lung injury. This study revealed that a decrease in PPAR-γ protein expression and an increase in NF-κB activation occurred in LPS-induced acute lung injury in vivo [162]. In an asthma model induced by OVA, rosiglitazone alleviated airway inflammation by reducing peribronchiolar inflammatory cell infiltration, goblet cell hyperplasia, and mucus secretion. These effects were attributed to the inhibition of both NF-κB expression and the activation of the toll-like receptor 2 (TLR2)/nod-like receptor with pyrin domain-containing 3 (NLRP3) inflammatory corpuscle pathway by the PPARγ agonist [163]. Furthermore, accumulating evidence suggests that rosiglitazone exerts anti-inflammatory effects by upregulating HO-1 in the pulmonary system [47, 108, 164, 165], indicating a potential therapeutic strategy for inflammatory diseases [43]. Our previous study demonstrated that rosiglitazone suppressed LPS-induced nuclear translocation of phosphorylated NF-κB (p65) and the expression of adhesion molecules through PPARγ-dependent HO-1 upregulation. Additionally, we found that rosiglitazone inhibited LPS-induced lung inflammation through alternative PPARγ-independent HO-1 induction [62]. Consequently, our results indicate that rosiglitazone-induced HO-1 expression was mediated by activation of the Nrf2 or PPARγ cascade and suppressed inflammatory responses triggered by LPS (Fig. 2). The positive effects of TZDs on attenuating inflammatory responses by upregulating HO-1 suggest that rosiglitazone could be a potent treatment for pulmonary inflammatory diseases [45, 160, 166].

Beyond their well-established anti-inflammatory actions, PPAR agonists, particularly PPARγ agonists like rosiglitazone, play a significant role in the resolution phase of inflammation, a process crucial for restoring tissue homeostasis [167]. This phase involves not just the suppression of proinflammatory signals but also actively promotes reparative and restorative processes in inflamed tissues. PPARγ agonists contribute to the resolution of inflammation through multiple mechanisms. They promote the polarization of macrophages towards the M2 phenotype, which are anti-inflammatory and essential for tissue repair and resolving inflammation [168]. These M2 macrophages play a crucial role in efferocytosis, the phagocytic clearance of apoptotic cells, preventing secondary necrosis and further inflammation. Additionally, PPARγ agonists suppress the expression of proinflammatory mediators like cytokines and chemokines and activate anti-inflammatory signaling pathways, including the inhibition of NF-κB and AP-1 [169]. These actions help in controlling the balance between pro- and anti-inflammatory signals. Furthermore, PPARγ agonists modulate the recruitment of immune cells to inflammation sites by regulating the expression of adhesion molecules and chemokines, thus influencing the immune cell population towards an anti-inflammatory environment [170]. The study by Lea et al. in the European Respiratory Journal in 2014 underscores these findings, demonstrating that rosiglitazone, a PPARγ agonist, increases M2 macrophage polarization and enhances efferocytosis in COPD alveolar macrophages. The study showed that COPD alveolar macrophages exhibit a skewed M2 phenotype and that treatment with rosiglitazone inhibited LPS-induced TNF-α and CCL-5 production [160]. It also increased the expression of CD36, HO-1, and PPARγ, key elements in the phagocytosis of apoptotic neutrophils and the resolution of inflammation [171]. In a subchronic tobacco smoke mouse model, rosiglitazone significantly reduced inflammatory cells in bronchoalveolar lavage, further illustrating its role in resolving pulmonary inflammation [172]. These findings collectively indicate that PPARγ agonists like rosiglitazone play a critical role in resolving inflammation by modulating macrophage function and enhancing efferocytosis, contributing to the restoration of pulmonary homeostasis and offering potential therapeutic avenues for chronic pulmonary diseases.

## Discussion

Understanding the mechanisms governing pulmonary inflammation is paramount in determining effective therapeutic strategies for diseases such as asthma, COPD, acute lung injury, and ARDS. This review examined the role of PPAR agonists, specifically PPARα, PPARβ, and PPARγ, in combating inflammatory and oxidative stress cascades in various pulmonary disorders. PPARα, PPARβ, and PPARγ exhibit differential tissue expression, indicating their involvement in different physiological functions. PPAR activation by endogenous and synthetic ligands can modulate inflammatory responses in the lungs. PPAR agonists inhibit crucial inflammatory mediators by disrupting pathways such as NF-κB, AP-1, and STATs. By interrupting these pathways, PPAR agonists exert potent anti-inflammatory effects, thereby halting chronic inflammation in the lungs. Pulmonary inflammation is characterized by oxidative stress. PPARs, especially PPARγ, regulate oxidative stress by modulating the NOX/ROS pathway. Additionally, PPAR agonists enhance antioxidant defenses, such as HO-1, thereby protecting against oxidative damage. PPAR agonists, with their dual actions against inflammation and oxidative stress, offer a promising therapeutic approach for pulmonary diseases. Synthetic PPAR agonists, which allow for precise dose control, have potential as future treatments. Clinical translation of PPAR agonists requires addressing safety concerns, patient variability, and long-term effects. Rigorous trials are essential for establishing optimal doses and safety profiles. Exploring novel delivery methods and combination therapies, as well as identifying predictive biomarkers, is crucial for future advancements. The balance between inflammation and oxidative stress in pulmonary diseases necessitates comprehensive therapeutic strategies. PPAR agonists, with their multifaceted effects, represent a promising avenue for managing these conditions. Ongoing research and clinical exploration play pivotal roles in unlocking the potential of PPAR agonists and present novel possibilities for patients grappling with these complex disorders.

In this comprehensive review, we showed a complex web of signaling pathways and transcription factors that orchestrate the protective effects of PPARγ agonists against pulmonary inflammation. The multifaceted nature of this regulation underscores the potential of targeting HO-1 expression as a therapeutic strategy for managing inflammatory lung diseases. PPARγ, which is a nuclear receptor with diverse physiological functions, stands at the nexus of this regulatory network [34, 35]. Its activation by rosiglitazone not only induces HO-1 expression but also mitigates LPS-mediated inflammatory responses [62], demonstrating the dual protective effects of PPARγ agonists against pulmonary inflammation. Our study shows that rosiglitazone-induced HO-1 expression occurs through both PPARγ-dependent and PPARγ-independent pathways, underscoring the complexity of PPARγ-mediated responses. While the PPARγ-dependent pathway involves Nrf2 activation and subsequent binding to ARE sites in the HO-1 promoter, the PPARγ-independent pathway, intriguingly, does not rely on canonical mediators such as NOX/ROS, c-Src/Pyk2, Akt, or Nrf2. These findings challenge existing paradigms and emphasize the need for further investigations to unravel the precise molecular mechanisms driving PPARγ-independent responses in pulmonary epithelial cells. On the other hand, the overexpression of HO-1 in cells can have several negative consequences, necessitating strict regulatory mechanisms to maintain a balanced cellular environment [173]. Firstly, excessive HO-1 activity can disrupt cellular homeostasis. This enzyme plays a key role in the degradation of heme into biliverdin, carbon monoxide, and free iron. While these byproducts have protective roles at moderate levels, their overproduction can lead to toxicity and disrupt various cellular processes, including oxidative stress responses and iron homeostasis [174]. Secondly, the accumulation of HO-1 byproducts such as carbon monoxide and biliverdin can be harmful. Carbon monoxide, although beneficial in small quantities as a signaling molecule, can be toxic at high concentrations, potentially impairing mitochondrial function and cellular respiration [175]. Biliverdin and its subsequent conversion to bilirubin are known for their antioxidant properties, but in excess, they can contribute to cellular stress and damage [174]. Finally, excessive HO-1 expression may trigger cytotoxic effects. While HO-1 is generally considered cytoprotective, primarily due to its role in reducing oxidative stress, its overexpression could paradoxically lead to cellular damage [176]. This could be due to an imbalance in prooxidant and antioxidant activities, leading to oxidative stress, or the dysregulation of other cellular pathways such as apoptosis or inflammation. In summary, while HO-1 is crucial for cellular protection against oxidative stress, its overexpression must be carefully regulated to prevent disturbances in cellular homeostasis, toxic accumulation of its byproducts, and potential cytotoxic effects. This underscores the importance of a balanced expression of HO-1 for maintaining cellular health and function.

Our study highlights Nrf2 as a pivotal mediator of rosiglitazone-induced HO-1 expression, particularly in the context of PPARγ-independent pathways [62]. Nrf2, which is activated in response to oxidative stress, controls the expression of antioxidant genes, including HO-1, by binding to ARE sites [50, 51]. Rosiglitazone enhances Nrf2 binding to the ARE site in the HO-1 promoter, verifying its role in mediating HO-1 induction. This Nrf2-mediated response is an attractive target for therapeutic interventions, and Nrf2 activation not only induces HO-1 but also regulates many antioxidant and cytoprotective genes, offering comprehensive cellular defense against oxidative insults. The interplay between SIRT1, PGC1α, and HO-1 is a crucial mechanism in PPARγ agonist-induced responses [127, 130]. Rosiglitazone-induced SIRT1 phosphorylation leads to the deacetylation of PGC1α, facilitating its translocation to the nucleus [70]. Inside the nucleus, PGC1α interacts with PPARγ, resulting in the phosphorylation of PPARγ. Additionally, a reduction in NCoR levels in the nucleus further enhances PPARγ phosphorylation. This interplay underscores the nuanced regulation of HO-1 expression, highlighting the cross-talk between various transcriptional regulators in the cellular response to PPARγ agonists. Sp1 and AP-1, which are transcription factors that are sensitive to oxidative stress and inflammatory signals, modulate HO-1 expression [147, 148, 150, 151]. Sp1, which is a central mediator of cellular responses, responds to a plethora of signals, orchestrating a finely tuned regulatory network [141, 142]. Our study showed the pivotal role of Sp1 in PPARγ agonist-induced HO-1 expression, offering a potential target for therapeutic interventions. Similarly, AP-1, which is composed of diverse family members, serves as a central player in cellular signaling, responding to oxidative and inflammatory stimuli [147, 148, 150]. Deciphering the interactions between AP-1 and PPARγ agonists provides insights into stress-responsive gene expression, paving the way for innovative strategies to mitigate oxidative stress-related disorders.

This comprehensive understanding of the molecular intricacies of PPARγ agonist-induced HO-1 expression opens exciting avenues for future research and therapeutic interventions. Further investigations into PPARγ-independent pathways to delineate the roles of unexplored mediators hold the key to identifying novel therapeutic targets. Additionally, exploring the interplay between different transcription factors and coregulators in the context of HO-1 regulation opens promising avenues for targeted therapies against pulmonary inflammatory diseases. Our previous studies provide a detailed roadmap of the signaling pathways and transcriptional regulators involved in PPARγ agonist-induced HO-1 expression in pulmonary alveolar epithelial cells. These findings not only deepen our understanding of the molecular mechanisms governing pulmonary inflammation but also offer potential targets for innovative therapeutic strategies. As we continue to dissect the complexities of these regulatory networks, we move closer to revolutionizing our approach to combating pulmonary inflammatory disorders, ultimately improving the quality of life of patients worldwide.

In this review, we underscored the potential of TZDs, which are PPARγ agonists, in shedding light on the molecular mechanisms governing HO-1 expression for the prevention and treatment of lung and airway inflammatory diseases (Fig. 2). The upregulation of HO-1 by rosiglitazone is mediated by both PPARγ-dependent and PPARγ-independent pathways in HPAEpiCs. In the PPARγ-dependent pathway, rosiglitazone enhances HO-1 expression by activating a cascade involving PKCα, AMPKα, p38 MAPKα, SIRT1, Ac-PGC1α deacetylation, NCoR fragmentation, and the direct binding of activated PPARγ to the HO-1 promoter’s responsive element. Conversely, in the PPARγ-independent pathway, rosiglitazone-induced HO-1 expression occurs via NOX/ROS-dependent phosphorylation of c-Src/Pyk2/Akt, leading to Nrf2 activation and its binding to the ARE region of the HO-1 promoter. Upregulating HO-1 exerts anti-inflammatory effects, particularly affecting the expression of adhesion molecules such as ICAM-1 and VCAM-1, which are associated with monocyte/leukocyte accumulation in pulmonary resident cells challenged with LPS. These findings highlight a novel approach for managing inflammatory pulmonary disorders and offers potential solutions to the increasing burden of chronic lung diseases worldwide.

*Note: The pathways depicted in the figure are adapted from previous studies* *[*62*,*^70^*]* *and suggest that enhancing HO-1 by using TZDs is a promising strategy for addressing the challenges posed by chronic lung diseases globally.*

## Abbreviations

AMPKα: 5′-adenosine monophosphate-activated protein kinase α
AP-1: activator protein 1
ARDS: acute respiratory distress syndrome
AREs: antioxidant response elements
ATP: adenosine-5′-triphosphate
cAMP: cyclic adenosine monophosphate
COPD: chronic obstructive pulmonary disease
COX-2: cyclooxygenase-2
cPLA_2_: cytosolic phospholipase A_2_
CREB: cAMP response element-binding protein
CBP: CREB-binding protein
CSE: cigarette smoke extract
DAG: diacylglycerol
EGFR: epidermal growth factor receptor
ERKs: extracellular signal-regulated protein kinases
GSK: glycogen synthase kinase
HO-1: heme oxygenase-1
HPAEpiCs: human pulmonary alveolar epithelial cells
ICAM-1: intercellular adhesion molecule-1
IL-1β: interleukin-1β
JNKs: c-Jun NH2-terminal kinases
LBD: ligand-binding domain
LPS: lipopolysaccharide
LTA: lipoteichoic acid
MAPKs: mitogen-activated protein kinases
MMP-9: matrix metalloproteinase-9
NCoR: nuclear receptor co-repressor
NF-κB: nuclear factor κ-light-chain-enhancer of activated B cells
NLRP3: nod-like receptor with pyrin domain containing 3
NOX: NADPH oxidase
Nrf2: nuclear factor E2-related factor 2
OVA: ovalbumin
PDGFR: platelet-derived growth factor receptor
PGC-1α: peroxisome proliferator-activated receptor gamma coactivator 1-α
PGE_2_: prostaglandin E_2_
PGJ_2_: prostaglandin J_2_
PKC: protein kinase C
PPAR: peroxisome proliferator-activated receptor
PPRE: peroxisome proliferator-activated receptor-response elements
Rac: Ras-related C3 botulinum toxin substrate
Redox: reduction-oxidation
REs: regulatory elements
ROS: reactive oxygen species
RXR: 9-cis retinoic acid receptor
siRNA: small interfering RNA
SIRT1: silent information regulator type-1
SRC-1: steroid receptor coactivator 1
STAT: signal transducer and activator of transcription protein
TF: transcription factor
TLR2: toll-like receptor 2
TNF-α: tumor necrosis factor-α
TZDs: thiazolidinediones
VCAM-1: vascular cell adhesion molecule-1
